# Compliance of procalcitonin-guided antibiotic therapy in adult infectious patients in China: a multicenter real-world retrospective study

**DOI:** 10.3389/fmed.2026.1769810

**Published:** 2026-05-18

**Authors:** Xiaojing Li, Anke Shi, Yu Wang, Xiaolan Chen, Guoqiang Zhang, Shengtao Yan

**Affiliations:** 1 Emergency Department, Peking University First Hospital, Beijing, China; 2 China-Japan Friendship Hospital (Institute of Clinical Medical Sciences), Chinese Academy of Medical Sciences & Peking Union Medical College, Beijing, China; 3 Capital Medical College, Beijing, China; 4 Emergency Department, China-Japan Friendship Hospital, Beijing, China

**Keywords:** antibiotic discontinuation, compliance, PCT-guided antibiotic use, procalcitonin, real-world study

## Abstract

**Introduction:**

Procalcitonin (PCT) is widely applied to guide antibiotic therapy, and its use—particularly for guiding antibiotic discontinuation—has been recommended in Chinese expert consensus for the past 5 years, in alignment with international guidelines. However, real-world adherence and optimal timing remain unclear.

**Methods:**

This retrospective study analyzed adult patients with confirmed infections admitted to emergency intensive care units (EICUs) in 2023. Compliance with guidelines was assessed, focusing on stopping antibiotics when PCT dropped below 0.5 ng/mL or decreased by ≥80% within 24, 48, and 72 h after meeting criteria.

**Results:**

Among 195 patients, 52.3% met PCT criteria had a lower mortality rate than non-compliant cases (13.7% vs. 28.0%, *p* = 0.023). Early discontinuation within 24 h occurred in18.6% and was associated with shorter EICU stays (7 vs. 18 days), shorter antibiotic durations (6 vs.14 days), and lower antibiotic density (5.0 vs. 22.0 DDDs) without increasing mortality. Similar trends were observed when 48 and 72 h were used as cutoffs.

**Discussion:**

The adherence to PCT-guided algorithms in routine practice in China—particularly timely discontinuation within 24 h—was low. Earlier discontinuation within 72 h was associated with shorter EICU stays and reduced antibiotic exposure. Importantly, no increase in mortality was observed. Further prospective studies are needed to confirm these findings.

## Introduction

In recent years, the resistance to commonly used antimicrobial agents is still on the rise for clinical bacterial isolates in China, leading to the increasing prevalence of multidrug-resistant bacteria and more challenging treatment as well as a heavier medical burden (1). The course of antibacterial therapy generally relies on empirical judgment and requires a comprehensive evaluation in combination with the type of pathogenic bacteria, drug resistance status, severity of infection and the response to treatment. However, it is not clear whether prolonged antibiotic treatment courses can reduce the failure and recurrence rates, but are one of the primary causes of resistance (2). Thus, determining the appropriate timing for discontinuation of antibiotics treatment remains a huge challenge in clinical practice.

Procalcitonin (PCT) is a biomarker that can be upregulated during systemic inflammatory responses driven by pathogen-associated molecular patterns (PAMPs) and damage-associated molecular patterns (DAMPs) (3). It has been widely used as an important reference indicator for the diagnosis and management of bacterial infections. This activation mechanism is consistent with the inflammatory cascade observed in sepsis (4). The prognostic value of a single PCT test is relatively limited, but a dynamic trend of sustained increase in PCT concentration usually indicates a poor prognosis for patients with severe sepsis or septic shock (5). Moreover, PCT holds significant clinical value in guiding the adjustment and discontinuation of antibiotic therapy for critically ill and septic patients. For intensive care units (ICU) patients with severe infections who are receiving antibiotic therapy, serial measurement of PCT concentrations can serve as a surrogate marker to facilitate early discontinuation of antibiotics, using thresholds such as a PCT concentration < 0.5 ng/mL or a reduction of ≤80% from peak values (6, 7). Previous studies have demonstrated that PCT-guided antibiotic therapy reduced the use of antibacterial drugs and shortened durations of mechanical ventilation and ICU stays, thereby resulting in a reduction of overall treatment costs (8–10). Moreover, recent meta-analyses have concluded that PCT-guided antibiotic therapy appears to be safe, reduces antibiotic use and may improve survival outcomes (11). In patients with acute respiratory tract infections, a systematic Cochrane review has shown that the antibiotic treatment duration can be significantly shortened without compromising clinical outcomes using PCT-guided antibiotic therapy (12). Although multiple randomized clinical trials have demonstrated the efficacy of PCT-guided antibiotic therapy, PCT testing appears to be irregular, and compliance with the PCT protocol is relatively low in routine clinical practice (13).

To date, there is a lack of systematic evaluation of the PCT concentration in guiding the discontinuation of antibiotic therapy in China, especially in a real-world setting. Moreover, while Chinese expert consensus recommends monitoring PCT concentrations every 24 h for critically ill patients with infectious (14), the optimal time frame for PCT-guided discontinuation and real-world adherence in China remain unclear. Therefore, using VIDAS BRAHMS PCT, we conducted a retrospective screening of confirmed adult infectious patients in the EICUs at Peking University First Hospital and the China-Japan Friendship Hospital from January to December 2023. The study aims were to clarify discrepancies between current clinical practices, and to explore the clinical application of the recommended stopping criteria for PCT concentrations and the frequency of PCT monitoring in China.

## Materials and methods

### Study design and participants

A retrospective screening of confirmed adult patients with infections admitted to the emergency intensive care units (EICUs) in Peking University First Hospital and the China-Japan Friendship Hospital was conducted from January to December 2023. For the diagnosis of infection, clinicians made comprehensive assessments based on pathogen identification results provided by microbiology specialists, combined with the suspected site of infection and other relevant clinical and laboratory findings. The study included adult patients who were admitted to the EICU for at least 3 days, had at least one PCT measurement of 0.5 ng/mL or higher, had complete clinical information and were definitively diagnosed with a bacterial infection. Patients were excluded from the study if they lacked a distinct diagnosis of infection or definite treatment outcomes, or did not receive antibiotic treatment during their hospitalization, or were subjected to long-term mechanical ventilation (defined as ≥21 consecutive days of invasive mechanical ventilation for ≥6 h per day) (15). Furthermore, patients in the following non-infectious conditions known to cause elevated PCT concentration were excluded: immunodeficiency (patients receiving chronic or high-dose immunosuppressive therapy) and a variety of systemic inflammatory diseases, renal insufficiency, malignant tumors, and transplantation-related immune reactions (5). The conditions that may have affected their short-term survival (end-of-life or treatment-limiting scenarios) were not included. Patients who required long-term antibiotic therapy for infections, including but not limited to infective endocarditis, post-surgical infections/local drainage after cardiothoracic surgery, open wounds and abscesses of the liver and brain, were also excluded.

In China, the use of antimicrobial agents is governed by a hierarchical stewardship system. In both participating EICUs, attending physicians are authorized to prescribe antibiotics within the restricted-use category. For special or highly regulated antimicrobial agents, a mandatory consultation system is implemented, requiring approval and guidance from designated clinical pharmacists. These pharmacists provide 24/7 support, ensuring that patients receive timely and appropriate antimicrobial therapy.

The study was approved by the ethical committees of Peking University First Hospital (approval number 2025YAN098-0002) and the China-Japan Friendship Hospital (approval number 2024-KY-417-1). All patients provided written informed consent.

### Data collection and endpoints

Data were collected with regard to patient age, gender, medical history, concomitant disease, SOFA, diagnosis of infection and the infection site(s), types of infectious microorganisms, laboratory test results and imaging results at the time of admission. Medical records were reviewed and the adherence rates to consensus-recommended criteria for PCT use were assessed. In addition, PCT monitoring results and duration of antibiotic use during the EICU stay, length of stay in EICU, mortality and survival were also carefully recorded.

The primary endpoints were: adherence rates to the consensus-recommended criteria for PCT use; duration of antibiotic use; and length of stay in the EICU. The secondary endpoints included antibiotic use density, mortality rate and median survival (censored at 110 days). Antibiotic use density was defined as the cumulative daily defined dose (DDD) of all antibiotics used during each patient’s EICU stay, which was the total amount of antibiotics administered per patient.

### Definition of adherence to the PCT protocol and grouping

In accordance with the expert consensus (6, 7), for adult patients with infections admitted to the EICU, empirical antibiotic treatment was administered while PCT was dynamically monitored. It is recommended to adopt a PCT monitoring frequency every 24–48 h for the reevaluation of the necessity of antibiotic treatment. Blood samples were collected and measurement of PCT concentrations were performed using highly sensitive immunoassays VIDAS^®^B. R. A. H. M. S. PCT kits (BioMérieux SA, France). For the recommendation of PCT stopping advice, it is suggested to cease antibiotic treatment when the PCT concentration drops below 0.5 ng/mL or experiences an 80% reduction from peak values.

Comparisons regarding baseline and clinical characteristics were performed between patients with PCT indication and patients without PCT indication:

(1) Patients with a PCT indication were defined as those who discontinued antibiotic treatment by adhering to the above PCT stopping criteria.

(2) Patients without PCT indications were defined as those in whom the PCT concentration did not exhibit an 80% downward trend during the monitoring period or had not yet reached an 80% reduction, but whose antibiotics treatment had been prematurely discontinued for clinical reasons. Additionally, PCT daily monitoring was not mandatory for patients without PCT indications in the real-world setting.

To explore the adherence rate of antibiotic discontinuation on the actual time window after reaching the PCT stopping advice, patients with PCT indications were assessed using 3 predefined time cutoffs (24, 48, and 72 h). For each cutoff, separate dichotomous comparisons of baseline and clinical characteristics were performed between patients who discontinued antibiotics within the specified time and those who discontinued at or beyond that time.

In addition, the current clinical status of patients who discontinued antibiotics when their PCT concentrations were <0.5 ng/mL and decreased by 80% from peak values are described, respectively. Among patients with PCT indications, if antibiotics were discontinued upon reaching a PCT concentration < 0.5 ng/mL or if patients simultaneously fulfilled the conditions of PCT < 0.5 ng/mL and ΔPCT > 80% from peak values, patients were classified into the PCT < 0.5 ng/mL group, while the remaining patients were classified into the ΔPCT > 80% group.

### Statistical analysis

All statistical analyses were performed using R software (version 4.3.3, R Foundation for Statistical Computing, Vienna, Austria). Except for age, which was expressed as the mean with standard deviation (SD), all other continuous variables are presented as the median and interquartile range (IQR). All categorical variables are given as numbers with percentage. A *p*-value < 0.05 was considered to be a significant difference. Categorical variable data were compared using the Chi-squared test, while continuous variables were compared using the Wilcoxon rank sum test. Survival analysis was performed using a Kaplan–Meier curve. All endpoints were compared between the patients with and without PCT indications, as well as between the three predefined time cutoffs (24, 48, and 72 h) of patients with PCT indications. In addition, the clinical pictures of patients who discontinued antibiotics on PCT stopping advice with a PCT concentration < 0.5 ng/mL or ΔPCT > 80% from peak values are also described.

## Results

### Patients disposition and baseline characteristics

**Figure 1:**
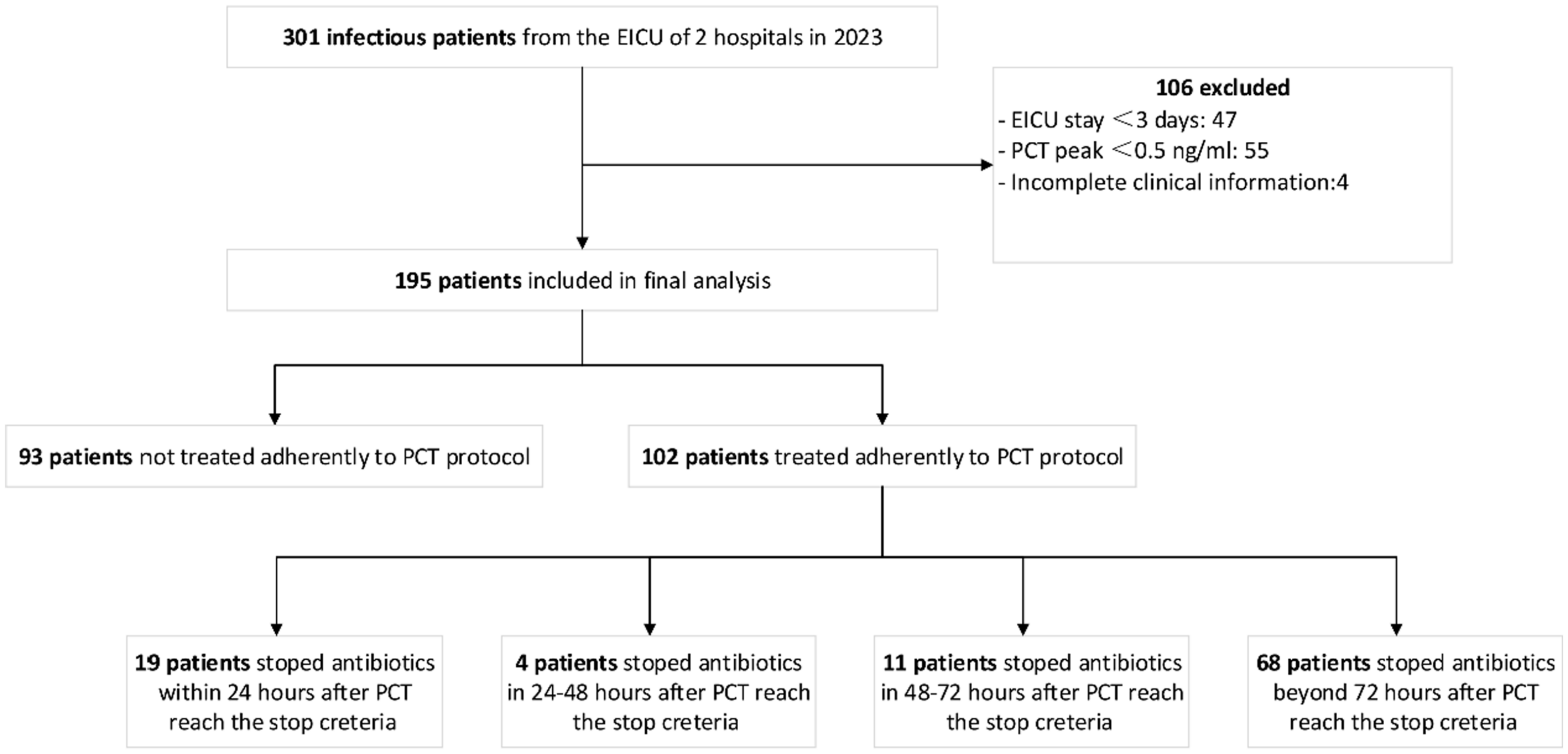
Disposition of the study patients.

The present study screened 301 patients with infections in the EICUs of two Chinese hospitals in 2023. Of these, 106 (35.2%) failed the screening for the following reasons: EICU stay < 3 days (47, 15.6%); PCT peak concentration < 0.5 ng/mL (55, 18.3%); and incomplete clinical information (4, 1.3%) (Figure 1). Finally, a total of 195 patients were included in the analysis, with 102 (52.3%) being treated with adherence to the PCT protocol and 93 (47.7%) not treated in this manner. Among 102 patients treated with adherence to the PCT protocol, 19 ceased treatments with antibiotics within 24 h of PCT concentrations reaching the stop criteria, 4 between 24 and 48 h, 11 between 48 and 72 h, and 68 stopped antibiotics at or beyond 72 h after PCT reached the stop criteria (Figure 1). The mean age of all enrolled patients was 67.0 years, with 35.9% being female. Patients had a median [IQR] SOFA of 8.0 [6.0–10.0]. The majority of patients (169, 86.7%) had lung infections, followed by abdominal infections (29, 14.9%). Among all enrolled patients, the predominant types of infectious microorganisms were Gram-negative bacteria (52.3%), followed by Gram-positive bacteria (33.8%) and fungi (31.8%). All enrolled patients had median initial and peak PCT concentrations of 2.1 ng/mL [IQR 0.5–11.8] and 6.6 ng/mL [IQR 1.7–20.0], respectively (Table 1).

**Table 1:** Baseline and clinical characteristics of patients with and without PCT indications.

Clinical characteristics	Total (*n* = 195)	Patients without PCT indication (*n* = 93)	Patients with PCT indication (*n* = 102)	*p*-value
Age (years), mean (SD)	67.0 (16.8)	66.8 (17.0)	67.2 (16.6)	0.898
Female, *n* (%)	70 (35.9)	31 (33.3)	39 (38.2)	0.573
SOFA, median [IQR]	8.0 [6.0, 10.0]	8.0 [6.0, 10.0]	8.00 [6.0, 11.0]	0.952
Initial PCT value (ng/mL), median [IQR]	2.1 [0.5, 11.8]	1.1 [0.3, 7.2]	2.5 [0.7, 14.4]	0.029
Peak PCT value (ng/mL), median [IQR]	6.6 [1.7, 20.0]	5.0 [2.0, 20.0]	9.4 [1.6, 21.0]	0.475
PCT testing frequency, median [IQR]	0.75 [0.50, 0.93]	0.75 [0.50, 1.00]	0.79 [0.50, 0.91]	0.780
Infection sites, *n* (%)				
Lung	169 (86.7)	84 (90.3)	85 (83.3)	0.221
Abdominal	29 (14.9)	11 (11.8)	18 (17.6)	0.348
Urinary system	16 (8.2)	9 (9.7)	7 (6.9)	0.650
Bloodstream	1 (0.5)	0 (0.0)	1 (1.0)	1.000
Intestine	2 (1.0)	1 (1.1)	1 (1.0)	1.000
Skin	5 (2.6)	3 (3.2)	2 (2.0)	0.917
Soft tissue of the skin	1 (0.5)	0 (0.0)	1 (1.0)	1.000
Mouth floor	1 (0.5)	1 (1.1)	0 (0.0)	0.963
Infectious microorganisms, *n* (%)				
Gram-positive bacterium	66 (33.8)	34 (36.6)	32 (31.4)	0.540
Gram-negative bacterium	102 (52.3)	41 (44.1)	61 (59.8)	0.040
Fungal	62 (31.8)	19 (20.4)	43 (42.2)	0.002
Others	9 (4.6)	8 (8.6)	1 (1.0)	0.028
No microbial	36 (18.5)	19 (20.4)	17 (16.7)	0.623
Missing	29 (14.9)	19 (20.4)	10 (9.8)	0.060
Mechanical ventilation, n (%)	128 (65.6)	61 (65.6)	67 (65.7)	1.000
Mortality rate, n (%)	40 (20.5)	26 (28.0)	14 (13.7)	0.023
EICU stay (days), median [IQR]	11.0 [6.0, 19.5]	6.0 [5.0, 14.0]	16.0 [10.0, 23.0]	< 0.001
Duration of antibiotic use (days), median [IQR]	10.0 [5.0, 14.0]	5.0 [3.0, 10.0]	14.0 [9.3, 18.0]	< 0.001
Antibiotic use density (DDDs), median [IQR]	8.6 [3.2, 23.5]	4.2 [2.0, 11.4]	17.4 [6.6, 30.0]	< 0.001

*p*-values for categorical variables were calculated using the chi-squared test. *p*-values for numerical variables were calculated using the Wilcoxon rank sum test.

DDD, sum of Daily Defined Dose; EICU, emergency intensive care units; IQR, interquartile range; PCT, procalcitonin; SD, standard deviation; SOFA, sequential organ failure score.

### The profiles of patients with and without PCT indications

**Figure 2:**
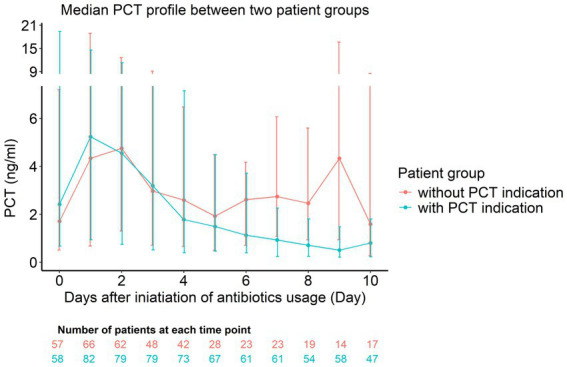
The trends of procalcitonin (PCT) concentrations in the patients with and without indications from the initiation of antibiotic treatment to 10 days. Data are presented as median PCT values (dots) with interquartile ranges (IQRs; vertical error bars) on the line chart.

**Figure 3:**
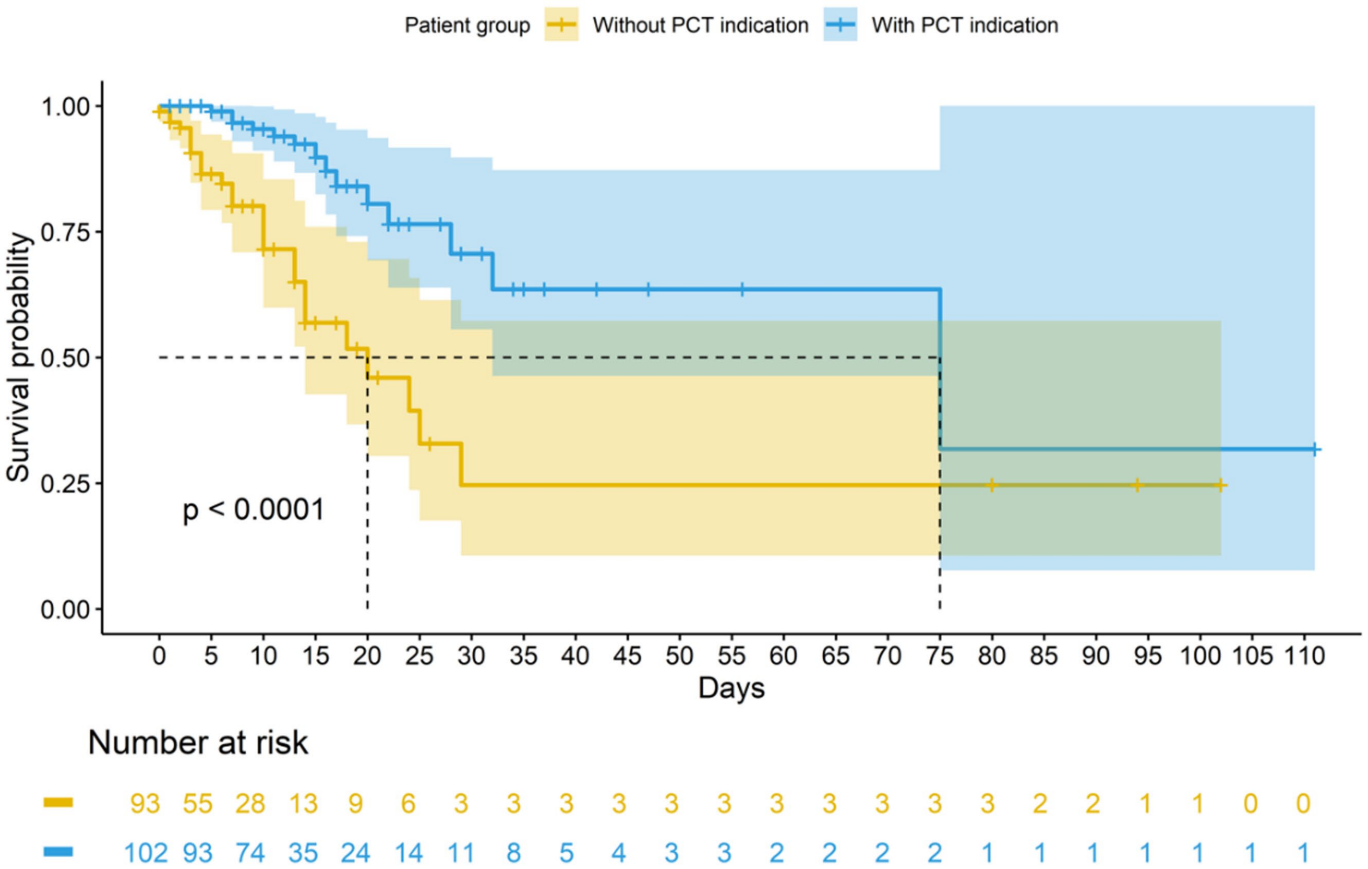
Kaplan–Meier survival curve (censored at 110 days) of patients with and without PCT indications.

Demographic characteristics, median SOFA, the percentage of patients with mechanical ventilation and infection sites were comparable between patients with and without PCT indications (all *p* > 0.05). The detection rates of Gram-negative bacteria (59.8% vs. 44.1%, *p* = 0.040) and fungal (42.2% vs. 20.4%, *p* = 0.002) infections were higher in patients with PCT indications compared to patients without PCT indications (Table 1). All fungal infections were mixed bacterial-fungal infections. The median values of initial PCT (2.5 vs. 1.1 ng/mL, *p* = 0.029) and peak PCT (9.4 vs. 5.0 ng/mL, *p* = 0.475) concentrations were higher in patients with PCT indications compared to patients without PCT indications, but statistically significance was only found for the initial PCT concentration. It is noteworthy that the median PCT concentration in patients with indications exhibited a sustained downward trend from the initiation of antibiotic treatment to 10 days. However, the median PCT concentration in patients without indications initially decreased but began to rise again from the 6th day of antibiotic treatment, showing no continuous decreasing trend (Figure 2). Regarding prognosis, patients with PCT indications had a lower mortality rate (13.7% vs. 28.0%, *p* = 0.023) and a prolonged median survival (75.0 vs. 20.0 days, *p* < 0.001) than patients without PCT indications (Table 1 and Figure 3). However, patients with PCT indications also experienced longer EICU stays (16.0 vs. 6.0 days, *p* < 0.001), duration of antibiotic use (14.0 vs. 5.0 days, *p* < 0.001), as well as a higher antibiotic use density (17.4 vs. 4.2 DDDs, *p* < 0.001) (Table 1).

### Profiles of patients with PCT indications according to the three-time windows

Demographic characteristics, median SOFA, median initial and peak PCT values, the percentage of patients with mechanical ventilation and infection sites, were comparable between adherent patients within each time window subgroup (all *p* > 0.05). The compliance rates were 18.6, 22.5 and 33.3% for patients who discontinued antibiotics within 24, 48, and 72 h after reaching the PCT stopping criteria, with most patients (66.7%) discontinuing antibiotic treatment at or beyond 72 h after reaching the PCT stopping criteria (Table 2). Patients who discontinued antibiotics treatment within 24 h upon reaching the PCT criteria (18.6%) had significantly shorter median EICU stays (7.0 vs. 18.0 days, *p* < 0.001), shorter antibiotic treatment durations (6.0 vs.14.0 days, *p* < 0.001), and lower antibiotic use density (5.0 vs. 22.0 DDDs, *p* < 0.001) compared to those who discontinued antibiotic treatment beyond 24 h (81.4%). Importantly, there was no difference in the mortality rates (15.8% vs. 13.3%, *p* = 1.000) between patients who discontinued antibiotics treatment within 24 h and those who did so after 24 h, after reaching the PCT stopping advice. Similar trends were observed when 48 and 72 h were used as cutoffs (Table 2).

**Table 2:** Baseline and clinical characteristics of patients with PCT indications according to the three-time windows (three separate dichotomous comparisons).

Clinical characteristics	Stop antibiotics (*n* = 102)								
	<24 h	≥24 h	*p*-value	<48 h	≥48 h	*p*-value	<72 h	≥72 h	*p*-value
Adherence rates, *n* (%)	19 (18.6)	83 (81.4)		23 (22.5)	79 (77.5)		34 (33.3)	68 (66.7)	
Age (years), mean (SD)	65.1 (19.1)	67.6 (16.1)	0.545	64.2 (18.1)	68.0 (16.2)	0.339	63.6 (16.7)	68.9 (16.4)	0.124
Female, *n* (%)	3 (15.8)	36 (43.4)	0.049	4 (17.4)	35 (44.3)	0.036	8 (23.5)	31 (45.6)	0.052
SOFA, median [IQR]	6.0 [5.5, 8.0]	8.0 [6.0, 11.0]	0.110	7.0 [5.5, 8.5]	8.0 [6.0, 11.5]	0.139	7.0 [6.0, 9.0]	8.0 [5.0, 11.3]	0.540
Initial PCT value (ng/mL), median [IQR]	1.5 [0.6, 11.6]	2.8 [0.8, 15.3]	0.429	1.5 [0.6, 9.3]	3.1 [0.8, 17.9]	0.271	1.6 [0.6, 5.7]	4.0 [0.8, 20.0]	0.075
Peak PCT value (ng/mL), median [IQR]	5.6 [2.1, 17.4]	9.5 [1.7, 23.5]	0.661	5.6 [1.6, 15.9]	10.7 [1.9, 25.4]	0.285	4.6 [1.4, 13.9]	11.3 [2.3, 30.9]	0.060
Infection sites, *n* (%)									
Lung	15 (78.9)	70 (84.3)	0.820	19 (82.6)	66 (83.5)	1.000	29 (85.3)	56 (82.4)	0.925
Abdominal	4 (21.1)	14 (16.9)	0.922	4 (17.4)	14 (17.7)	1.000	5 (14.7)	13 (19.1)	0.783
Urinary system	0	7 (8.4)	0.419	0	7 (8.9)	0.312	1 (2.9)	6 (8.8)	0.489
Bloodstream	0	1 (1.2)	1.000	0	1 (1.3)	1.000	0	1 (1.5)	1.000
Intestine	0	1 (1.2)	1.000	0	1 (1.3)	1.000	0	1 (1.5)	1.000
Skin	0	2 (2.4)	1.000	0	2 (2.5)	1.000	1 (2.9)	1 (1.5)	1.000
Soft tissue of the skin	0	1 (1.2)	1.000	0	1 (1.3)	1.000	0	1 (1.5)	1.000
Infectious microorganisms, *n* (%)									
Gram-positive bacteria	3 (15.8)	29 (34.9)	0.177	4 (17.4)	28 (35.4)	0.166	7 (20.6)	25 (36.8)	0.152
Gram-negative bacteria	6 (31.6)	55 (66.3)	0.012	7 (30.4)	54 (68.4)	0.003	14 (41.2)	47 (69.1)	0.012
Fungal	3 (15.8)	40 (48.2)	0.020	5 (21.7)	38 (48.1)	0.044	10 (29.4)	33 (48.5)	0.103
Others	0	1 (1.2)	1.000	0 (0.0)	1 (1.3)	1.000	0 (0.0)	1 (1.5)	1.000
No microbial	6 (31.6)	11 (13.3)	0.111	7 (30.4)	10 (12.7)	0.090	9 (26.5)	8 (11.8)	0.110
Missing	5 (26.3)	5 (6.0)	0.024	6 (26.1)	4 (5.1)	0.010	7 (20.6)	3 (4.4)	0.025
Mechanical ventilation, *n* (%)	12 (63.2)	55 (66.3)	1.000	13 (56.5)	54 (68.4)	0.422	19 (55.9)	48 (70.6)	0.210
Mortality rate, *n* (%)	3 (15.8)	11 (13.3)	1.000	3 (13.0)	11 (13.9)	1.000	5 (14.7)	9 (13.2)	1.000
EICU stay (days), median [IQR]	7.0 [5.0, 10.0]	18.00 [12.0, 25.5]	<0.001	7.0 [5.5, 10.5]	18.0 [13.0, 26.5]	<0.001	9.00 [6.0, 13.5]	18.00 [13.8, 29.0]	<0.001
Duration of antibiotic use (days), median [IQR]	6.0 [4.5, 7.0]	14.0 [11.0, 20.0]	<0.001	6.0 [4.50, 7.5]	14.0 [12.0, 20.5]	<0.001	7.00 [5.0, 11.0]	14.00 [12.8, 21.0]	<0.001
Antibiotic use density (DDD), median [IQR]	5.0 [2.5, 10.1]	22.2 [10.4, 32.3]	<0.001	5.0 [2.5, 10.2]	22.2 [10.9, 32.6]	<0.001	9.3 [4.0, 13.5]	24.1 [11.6, 36.3]	<0.001

Time cutoffs were defined as hours after PCT reached the stopping criteria. *p*-values for categorical variables were calculated using the chi-squared test. *p*-values for numerical variables were calculated using the Wilcoxon rank sum test. DDD, sum of Daily Defined Dose; EICU, emergency intensive care units; IQR, interquartile range; PCT, procalcitonin; SD, standard deviation; SOFA, sequential organ failure score.

### Exploration of the current clinical status on the stopping criteria of PCT < 0.5 ng/mL or ΔPCT > 80%

Patients were divided into two groups based on two PCT stopping criteria: PCT < 0.5 ng/mL (*n* = 42) and ΔPCT > 80% (*n* = 60). The median values of the initial PCT (0.9 vs. 9.2 ng/mL, *p* < 0.001) and peak PCT (1.4 vs. 17.5 ng/mL, *p* < 0.001) concentrations of patients in the PCT < 0.5 group were significantly lower than those in the ΔPCT > 80% group; however, there was no significant difference in the median SOFA between the two groups. In clinical practice, regardless of which PCT stopping criteria were used, the majority of patients showed a high adherence to antibiotic discontinuation ≥72 h (61.9% vs. 70.0%, *p* = 0.393), with similar adherence rates to discontinuation <24 h (19.0% vs. 18.3%) (Table 3). Patients who discontinued antibiotic treatment based on a PCT concentration < 0.5 ng/mL appeared to have a slightly lower mortality rate compared to those in the ΔPCT > 80% group (7.1% vs. 18.3%, *p* = 0.106), although again this apparent difference was not statistically significant.

**Table 3:** Patient clinical pictures according to a stopping criterion of PCT < 0.5 or ΔPCT > 80%.

Clinical characteristics	PCT < 0.5 ng/mL (*n* = 42)	ΔPCT > 80% (*n* = 60)	*p*-value
Age (years), mean (SD)	66.9 (16.3)	67.3 (17.0)	0.912
Female, *n* (%)	16 (38.1%)	23 (38.3%)	0.981
SOFA, median [IQR]	7.0 [5.0, 10.0]	8.0 [6.0, 11.3]	0.130
Initial PCT value (ng/mL), median [IQR]	0.9 [0.5, 1.7]	9.2 [2.7, 22.6]	< 0.001
Peak PCT value (ng/mL), median [IQR]	1.4 [0.8, 2.8]	17.5 [9.5, 38.8]	< 0.001
Adherence rates according to the time windows, *n* (%)			
<24 h	8 (19.0)	11 (18.3)	0.927
24–48 h	2 (4.8)	2 (3.3)	0.715
48–72 h	6 (14.3)	5 (8.3)	0.340
≥72 h	26 (61.9)	42 (70.0)	0.393
Infectious microorganisms, *n* (%)			
Gram-positive bacteria	15 (35.7)	17 (28.3)	0.429
Gram-negative bacteria	23 (54.8)	38 (63.3)	0.385
G^+^/G^−^ mixed infection	0	15 (25.0)	< 0.001
Fungal	15 (35.7)	28 (46.7)	0.270
Organ failure, *n* (%)	11 (26.2)	16 (26.7)	0.957
Respiratory failure, *n* (%)	14 (33.3)	12 (20.0)	0.128
Mortality rate, *n* (%)	3 (7.1)	11 (18.3)	0.106

*p*-values for categorical variables were calculated using the chi-squared test. *p*-values for numerical variables were calculated using the Wilcoxon rank sum test.

IQR, interquartile range; PCT, procalcitonin; SD, standard deviation; SOFA, sequential organ failure score.

## Discussion

The Chinese guidelines for recommended daily PCT testing aim to guide the discontinuation of antibiotic treatment in critically ill patients with infections, but compliance in a real-world setting is rarely reported. To the best of our knowledge, the present study is the first real-world retrospective analysis of the current adherence status of PCT-guided antibiotic discontinuation in China. Our findings revealed that a PCT stopping criterion was reached in 102 (52.3%) patients, while 93 (47.7%) discontinued the antibiotic treatment before reaching the PCT stopping criteria. The observed adherence rate appears lower than would be expected given the long-standing promotion of the relevant guideline recommendations.

Given the substantial number of potential confounders inherent in real-world observational studies, we additionally performed confounding covariate balancing in our analysis. Based on the clinical relevance of each variable and its potential influence on outcomes or between-group imbalance, the following covariates were included: SOFA score, PCT testing frequency, initial PCT value, and the presence of Gram-positive bacteria, Gram-negative bacteria, and fungal pathogens. To address covariate imbalance between the treatment and control groups, we applied two weighting approaches—conventional inverse probability of treatment weighting (IPTW) and entropy balancing (EB) (details in Supplementary methods). After adjustment using these methods, the differences observed between the groups that stopped antibiotics versus without PCT indication remained directionally consistent (Supplementary Tables S1, S2). Previous studies have demonstrated that non-sustained declines of PCT concentrations (i.e., decreased by 80% of peak values) was an independent predictor of mortality, and PCT-guided antibiotic discontinuation was also associated with improved survival (11, 16). Consistent with previous studies, patients without PCT indication had a significantly higher mortality rate and a shorter median survival compared to those who adhered to the recommended PCT stopping criteria. In addition, the dynamic trend of PCT in patients without PCT indications was initially a decrease followed by an increase, which was different from the continuous decline trend in patients with PCT indications. As a result, the continuous decline of PCT over time indicated a better prognosis for patients with PCT indications, which were consistent with previous studies (17, 18). The PCT concentrations in patients without PCT indications exhibited significant fluctuations during the treatment course, likely attributable to the complexity of their conditions and the inefficacy of the antibiotic therapy, which may collectively indicate a poor prognosis in these patients. Interestingly, among patients without a PCT indication, a shorter EICU stay and lower antibiotic use density were observed. This seemingly paradoxical finding may be explained by their higher mortality rate, which could have led to earlier discontinuation of intensive care, thereby shortening antibiotic exposure.

In clinical practice, although the PCT stopping advice had been reached, antibiotic therapy still proceeded, resulting in lower adherence to the PCT algorithm in actual clinical practice than in randomized clinical trials (19). Moreover, as the illness intensifies, the adherence to the PCT algorithm was lower, potentially due to the complexity of real-world cases and physicians’ tendency to rely more on the self-perceived levels of experience and integrate multiple indicators rather than a single PCT concentration (20). In the present study, most patients (66.7%) discontinuing antibiotic treatment beyond 72 h after reaching the PCT stopping criteria. Accordingly, only 18.6% of patients discontinued antibiotics within 24 h, a markedly lower proportion than that reported in the SAPS trial, in which 44% of patients had antibiotics stopped within 24 h (8). Although the SAPS trial was a randomized controlled study and our analysis reflects real-world clinical practice, the observed difference in early discontinuation rates remains substantial. The results of our study also indicated that there was no significant difference in mortality of patients who discontinued antibiotic treatment within 24, 48, or 72 h after reaching the PCT stopping criteria and those who discontinued treatment at or beyond the corresponding time cutoffs. Nevertheless, patients who were early discontinued from antibiotic treatment based on the PCT algorithm showed shorter lengths of stay in the EICU and durations of antibiotic treatment, as well as reduced usage of antibiotics, results consistent with previous research findings (8, 13, 21–23)—although these associations should not be interpreted as causal given the potential for reverse causality (i.e., earlier clinical improvement may have enabled earlier discontinuation). Moreover, the EICU length of stay was shorter in PCT-guided antibiotic discontinuation group, which may possibly contribute to lower in hospital charges (21, 24). Therefore, clinicians are enabled to discontinue antibiotic treatment earlier in accordance with the PCT algorithm without affecting the treatment outcome, while reducing unnecessary antibiotic use, thereby resulting in a expense saving. These findings suggest that compliance with the PCT algorithm was currently relatively low in China and that there remains a considerable scope for improvement in the adherence to PCT protocols in clinical practice.

The guidelines proposed a PCT concentration < 0.5 ng/mL or an 80% decline from the peak value as the threshold for antibiotic treatment discontinuation (6, 7, 14). Nevertheless, clinicians often report that they were inclined to discontinue antibiotic treatment when PCT was <0.5 ng/mL to guarantee safety in clinical practice in China. Consequently, our study investigated the real clinical picture regarding these two stopping criteria: PCT < 0.5 ng/mL and ΔPCT > 80%, respectively. However, according to the results, when the PCT concentration was <0.5 ng/mL, clinicians did not discontinue the antibiotic treatment immediately, with most patients having discontinued antibiotic therapy more than 72 h (61.9% vs. 70.0%) after reaching the PCT stopping criteria of a PCT concentration < 0.5 ng/mL and ΔPCT > 80%, respectively. The adherence rates based on these two criteria were similar for discontinuation within 24, 24–48, and 48–72 h, which is not in line with the current understanding of clinicians. However, regardless of which PCT stopping criterion was adopted, there was no significant difference in the mortality rate. Previous studies have also shown that, for critically ill patients, relative variations of PCT concentration seemed to be more important than absolute values for the clinical decision to discontinue antibiotic treatment (13, 22). Therefore, in patients with high initial PCT concentration, a decline of PCT > 80% from peak values may represent a potentially more achievable indicator of treatment response, rather than a definitive criterion for antibiotic discontinuation. Of course, this approach will require further accumulation of clinical experience with PCT use among clinicians in China.

There were some limitations to the present study. First, as a real-world investigation, it is subject to numerous potential confounders; although we adjusted for confounding in the analyses, the inclusion of all relevant covariates was constrained by the availability of clinical information (such as antimicrobial susceptibility results during antibiotics therapy, comorbidities, organ failure beyond SOFA, immunosuppression). Second, the non-adherence group comprised patients who discontinued antibiotics for heterogeneous clinical reasons (e.g., clinician judgment, changes in goals of care, or early death), introducing potential selection bias. Third, the subgroup analysis based on time windows included a relatively small sample size (such as *n* = 19 for <24 h), and the retrospective design necessitates cautious interpretation of all the findings. Fourth, the study enrolled patients with infections in the ICU in a broad manner. Subsequent prospective studies need to further categorize patient types, such as specifically investigating the PCT-guide for antibiotic discontinuation in sepsis or secondary infection patients. Finally, our study lacks formal cost analyses, requiring further prospective research to evaluate cost-effectiveness and to validate the applicability of these results in this specific population of patients.

## Conclusion

In clinical practice in China, adherence to PCT-guided algorithms (especially within 24 h) was generally low and the duration of antibiotic therapy was long, indicating substantial room for improvement. Earlier discontinuation—within 72 h of meeting the stopping criterion—was associated with shorter EICU length of stay and reduced antibiotic use. No increase in mortality was observed. Adherence rates were comparable between the two stopping thresholds (PCT < 0.5 ng/mL vs. ΔPCT > 80%). Nevertheless, further prospective studies will be required to confirm these results.

## Data Availability

The raw data supporting the conclusions of this article will be made available by the authors, without undue reservation.
