# Supplementary material for: Compliance of procalcitonin-guided antibiotic therapy in adult infectious patients in China: a multicenter real-world retrospective study
Source: Front Med (Lausanne). 2026 May 18;13:1769810. doi: 10.3389/fmed.2026.1769810 (PMC13222826; doi:10.3389/fmed.2026.1769810)
Supplement: Supplementary file 1 [file Data_Sheet_1.pdf]

Supplementary Methods and Results

Supplementary Methods: Covariate Balancing and Diagnosis of Covariate Overlap (Assumption of Positivity)

This observational study evaluated the clinical impact of procalcitonin (PCT)-guided antibiotic discontinuation. Given the non-randomized nature of treatment assignment, potential confounding and baseline covariate imbalance were explicitly addressed using methods based on weighting.

Covariates selected for adjustment were based on a priori clinical relevance rather than data-driven statistical criteria. The following baseline variables were included: sex, age, Sequential Organ Failure Assessment (SOFA) score, PCT testing frequency, initial PCT concentration at admission, and the presence of Gram-positive, Gram-negative, and fungal infections.

Two weighting strategies were implemented and compared: inverse probability of treatment weighting (IPTW) based on the propensity score, and entropy balancing (EB). For IPTW, the propensity score was estimated using a multivariable logistic regression model incorporating the pre-specified baseline covariates. Continuous variables were entered as linear terms. Interaction terms and higher-order terms were not routinely included, as adequate balance was achieved without additional model complexity. The propensity score model was specified for confounding adjustment rather than treatment prediction; therefore, model discrimination metrics were not used. Stabilized weights were constructed based on propensity score accordingly.

Entropy balancing was applied to directly reweight patients in each group so that the specified covariate distributions (means and, where appropriate, higher moments) were exactly balanced between patients who discontinued antibiotics prior to meeting PCT stopping criteria and those who discontinued antibiotics thereafter.

Covariate balance after weighting was assessed using standardized mean differences (SMD). SMD below 0.1 was considered indicative of adequate balance. Based on balance diagnostics, entropy balancing achieved slightly superior covariate balance compared with IPTW and was therefore selected as the primary weighting approach for outcome analyses.

Adequacy of covariate overlap (the positivity assumption) was evaluated by inspecting the overlap of propensity score distributions and examination of the distribution of individual weights. Evidence of substantial overlap and the absence of extreme weights were considered indicative of appropriate positivity.

All statistical analyses were performed using R software (version 4,3,3) [1]. The R package *WeightIt* (version 1.3.0) [2] was used for both IPTW and entropy balancing analysis.

Supplementary Results:

Covariate Balance Diagnostics

Table S1 presents the baseline and clinical characteristics of patients before covariate balancing. Several variables, including Gram-negative bacterium infection, fungal infection, mortality rate, EICU length of stay, duration of antibiotic use, and antibiotic use density, demonstrated large standardized mean differences (SMDs > 0.3) between two groups, indicating substantial imbalance prior to adjustment.

Among these variables, mortality rate, EICU length of stay, duration of antibiotic use, and antibiotic use density are clinical outcome variables that should not be adjusted. Gram-negative bacterium infection and fungal infection were selected as target covariates for balancing. In addition, other clinically relevant variables—including sex, age, SOFA score, PCT values at admission, PCT test frequency, and Gram-positive infection—were also incorporated into the covariate balancing procedure based on clinical relevance.

Table S1. Baseline and clinical characteristics of patients with and without PCT indications before covariate balancing.

	**Total (n= 195)**	**Stopping without PCT indication (n= 93)**	**Stopping after PCT indication (n= 102)**	**SMD**	***P*-value**
Age (years), mean (SD)	67.0 (16.8)	66.8 (17.0)	67.2 (16.6)	0.018	0.898
Female, n (%)	70 (35.9)	31 (33.3)	39 (38.2)	0.102	0.573
SOFA, median [IQR]	8.0 [6.0, 10.0]	8.0 [6.0, 10.0]	8.00 [6.0, 11.0]	0.015	0.952
Initial PCT value (ng/mL), median [IQR]	2.1 [0.5, 11.8]	1.1 [0.3, 7.2]	2.5 [0.7, 14.4]	0.035	0.029
Peak PCT value (ng/mL), median [IQR]	6.6 [1.7, 20.0]	5.0 [2.0, 20.0]	9.4 [1.6, 21.0]	0.097	0.475
PCT testing frequency, median [IQR]	0.8 [0.5, 0.9]	0.8 [0.5, 1.0]	0.8 [0.5, 0.9]	0.020	0.780
Infection sites, n (%)					
Lung	169 (86.7)	84 (90.3)	85 (83.3)	0.208	0.221
Abdominal	29 (14.9)	11 (11.8)	18 (17.6)	0.165	0.348
Urinary system	16 (8.2)	9 (9.7)	7 (6.9)	0.102	0.650
Bloodstream	1 (0.5)	0 (0.0)	1 (1.0)	0.141	1.000
Intestine	2 (1.0)	1 (1.1)	1 (1.0)	0.009	1.000
Skin	5 (2.6)	3 (3.2)	2 (2.0)	0.080	0.917
Soft tissue of the skin	1 (0.5)	0 (0.0)	1 (1.0)	0.141	1.000
Mouth floor	1 (0.5)	1 (1.1)	0 (0.0)	0.147	0.963
Infectious microorganisms, n (%)					
Gram-positive bacterium	66 (33.8)	34 (36.6)	32 (31.4)	0.110	0.540
Gram-negative bacterium	102 (52.3)	41 (44.1)	61 (59.8)	0.319	0.040
Fungal	62 (31.8)	19 (20.4)	43 (42.2)	0.482	0.002
Others	9 (4.6)	8 (8.6)	1 (1.0)	0.363	0.028

No microbial	36 (18.5)	19 (20.4)	17 (16.7)	0.097	0.623
Missing	29 (14.9)	19 (20.4)	10 (9.8)	0.300	0.060
Mechanical ventilation, n (%)	128 (65.6)	61 (65.6)	67 (65.7)	0.002	1.000
Mortality rate, n (%)	40 (20.5)	26 (28.0)	14 (13.7)	0.356	0.023
EICU stay (days), median [IQR]	11.0 [6.0, 19.5]	6.0 [5.0, 14.0]	16.0 [10.0, 23.0]	0.302	<0.001
Duration of antibiotic use (days), median [IQR]	10.0 [5.0, 14.0]	5.0 [3.0, 10.0]	14.0 [9.3, 18.0]	0.464	<0.001
Antibiotic use density (DDDs), median [IQR]	8.6 [3.2, 23.5]	4.2 [2.0, 11.4]	17.4 [6.6, 30.0]	0.482	<0.001

p values for numerical variables calculated using Wilcoxon rank sum test if not explicitly specified,

p values for categorical variables calculated using Chi-squared test.

SMD, standardized mean difference.

Abbreviations: DDD, sum of Daily Defined Dose; EICU, emergency intensive care units; IQR, interquartile range; PCT, procalcitonin; SD, standard deviation; SOFA, sequential organ failure score.

Table S2 summarizes the weighted comparison of patient characteristics after applying entropy balancing, along with the corresponding SMDs. Across all prespecified covariates targeted for balancing, SMDs were below 0.1, indicating adequate balance following weighting. Although several covariates related to the site of infection exhibited SMDs greater than 0.1 after weighting, these variables were not specified as target covariates in the balancing procedure and were not considered major determinants of antibiotic discontinuation decisions from a clinical perspective. Consequently, residual imbalance in these variables does not suggest inadequate covariate balancing or insufficient control of confounding.

Table S2. Baseline and clinical characteristics of patients with and without PCT indications after confounding covariate balancing.

	**Total (n= 195)**	**Stopping without PCT indication (n= 93)**	**Stopping after PCT indication (n= 102)**	**SMD**	***P*-value**
Age (years), mean (SD)	67.0 (16.7)	67.7 (17.0)	66.2 (16.5)	0.088	0.559
Female, n (%)	71.0 (36.4)	32.6 (35.0)	38.4 (37.6)	0.053	0.725
SOFA, median [IQR]	8.0 [6.0, 10.0]	8.0 [6.0, 10.0]	8.0 [6.0, 10.0]	<0.001	0.879
Initial PCT value (ng/mL), median [IQR]	1.7 [0.5, 10.8]	0.9 [0.3, 7.2]	2.4 [0.7, 12.5]	<0.001	0.019
Peak PCT value (ng/mL), median [IQR]	6.4 [1.7, 20.0]	5.0 [1.9, 17.4]	8.6 [1.6, 20.0]	0.112	0.617
PCT testing frequency, median [IQR]	0.8 [0.5, 0.9]	0.8 [0.5, 1.0]	0.8 [0.5, 0.9]	<0.001	0.741
Infection sites, n (%)					
Lung	169.0 (86.7)	85.2 (91.7)	83.8 (82.1)	0.284	0.053
Abdominal	28.9 (14.8)	9.5 (10.2)	19.5 (19.1)	0.254	0.081
Urinary system	16.2 (8.3)	10.7 (11.5)	5.4 (5.3)	0.225	0.112
Bloodstream	0.7 (0.4)	0.0 (0.0)	0.7 (0.7)	0.119	0.343

Intestine	2.3 (1.2)	0.9 (1.0)	1.3 (1.3)	0.032	0.834
Skin	5.2 (2.7)	3.4 (3.7)	1.8 (1.8)	0.116	0.421
Soft tissue of the skin	0.9 (0.5)	0.0 (0.0)	0.9 (0.9)	0.135	0.341
Mouth floor	0.7 (0.4)	0.7 (0.7)	0.0 (0.0)	0.122	0.298
Infectious microorganisms, n (%)					
Gram-positive bacterium	66.0 (33.8)	31.5 (33.8)	34.5 (33.8)	<0.001	1.000
Gram-negative bacterium	102.0 (52.3)	48.6 (52.3)	53.4 (52.3)	<0.001	1.000
Fungal	62.0 (31.8)	29.6 (31.8)	32.4 (31.8)	<0.001	1.000
Others	11.4 (5.8)	10.3 (11.1)	1.1 (1.0)	0.431	0.005
No microbial	36.4 (18.7)	15.5 (16.7)	20.9 (20.5)	0.097	0.504
Missing	28.9 (14.8)	15.7 (16.9)	13.2 (12.9)	0.112	0.459
Mechanical ventilation, n (%)	129.5 (66.4)	65.4 (70.3)	64.1 (62.9)	0.158	0.284
Mortality rate, n (%)	41.1 (21.1)	28.7 (30.8)	12.5 (12.2)	0.464	0.002
EICU stay (days), median [IQR]	11.0 [6.0, 19.0]	7.0 [5.0, 16.0]	14.0 [10.0, 23.0]	0.142	<0.001
Duration of antibiotic use (days), median [IQR]	10.0 [5.0, 14.0]	5.2 [3.0, 13.0]	13.0 [7.3, 17.2]	0.290	<0.001
Antibiotic use density (DDDs), median [IQR]	8.7 [3.4, 23.4]	4.8 [2.0, 12.0]	15.2 [5.9, 28.0]	0.347	<0.001

p values for numerical variables calculated using Wilcoxon rank sum test if not explicitly specified,

p values for categorical variables calculated using Chi-squared test.

SMD, standardized mean difference.

Abbreviations: DDD, sum of Daily Defined Dose; EICU, emergency intensive care units; IQR, interquartile range; PCT, procalcitonin; SD, standard deviation; SOFA, sequential organ failure score.

Assessment of Covariate Overlap (Positivity Assumption)

The propensity scores for all patients range from 0.218 to 0.801. No patient scores close to zero. Figure S1 illustrates the distribution of propensity scores across the two groups. Substantial overlap was observed throughout the entire range of propensity scores, supporting adequate covariate overlap between groups.

Patient-level weights derived from the entropy balancing approach range from 0.551 to 2.095. No weight truncation or trimming was applied. Figure S2 displays the distribution of these weights, with no evidence of extreme or highly influential weights (e.g., weights exceeding 20). These findings further support satisfactory covariate overlap and fulfillment of the positivity assumption, consistent with the propensity score–based overlap assessment.

Figure S1. Distribution of propensity scores of the patients.

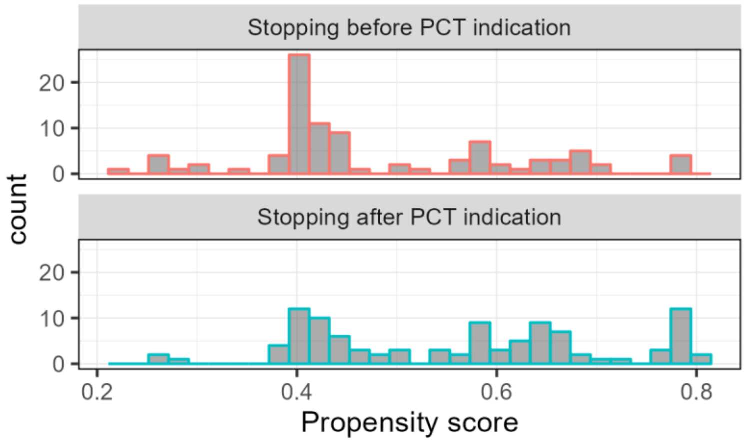

Figure S2. Distribution of patient-level weights derived from entropy balancing.

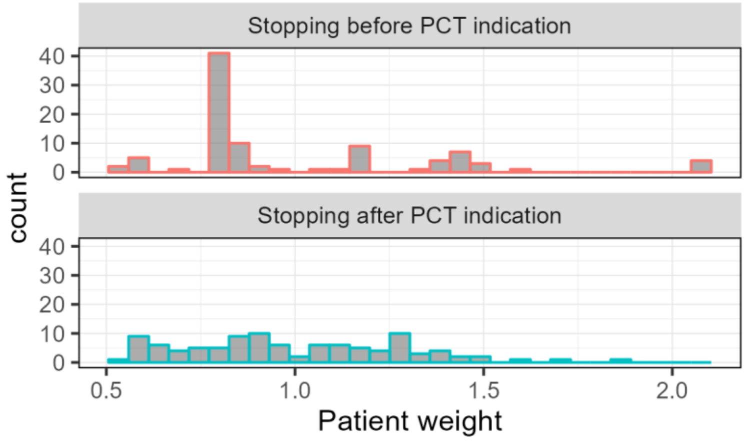

Overall, these diagnostics assessments support the adequacy of covariate balance, the satisfaction of the positivity (overlap) assumption, and the appropriateness of the selected weighting strategy for the present study.

References:

[1] R Core Team (2024). _R: A Language and Environment for Statistical Computing. R Foundation for Statistical Computing, Vienna, Austria. <https://www.R-project.org/>.

[2] Greifer N (2024). _WeightIt: Weighting for Covariate Balance in Observational Studies. R package version 1.3.0, <https://CRAN.R-project.org/package=WeightIt>.
